# The Myofibroblast Fate of Therapeutic Mesenchymal Stromal Cells: Regeneration, Repair, or Despair?

**DOI:** 10.3390/ijms25168712

**Published:** 2024-08-09

**Authors:** Fereshteh Sadat Younesi, Boris Hinz

**Affiliations:** 1 Faculty of Dentistry, University of Toronto, Toronto, ON M5G 1G6, Canada; fereshteh.younesi@mail.utoronto.ca; 2 Keenan Research Institute for Biomedical Science, St. Michael’s Hospital, Toronto, ON M5B 1T8, Canada

**Keywords:** fibrosis, skin, wound healing, mechanotransduction, epigenetics, mechanical memory

## Abstract

Mesenchymal stromal cells (MSCs) can be isolated from various tissues of healthy or patient donors to be retransplanted in cell therapies. Because the number of MSCs obtained from biopsies is typically too low for direct clinical application, MSC expansion in cell culture is required. However, ex vivo amplification often reduces the desired MSC regenerative potential and enhances undesired traits, such as activation into fibrogenic myofibroblasts. Transiently activated myofibroblasts restore tissue integrity after organ injury by producing and contracting extracellular matrix into scar tissue. In contrast, persistent myofibroblasts cause excessive scarring—called fibrosis—that destroys organ function. In this review, we focus on the relevance and molecular mechanisms of myofibroblast activation upon contact with stiff cell culture plastic or recipient scar tissue, such as hypertrophic scars of large skin burns. We discuss cell mechanoperception mechanisms such as integrins and stretch-activated channels, mechanotransduction through the contractile actin cytoskeleton, and conversion of mechanical signals into transcriptional programs via mechanosensitive co-transcription factors, such as YAP, TAZ, and MRTF. We further elaborate how prolonged mechanical stress can create persistent myofibroblast memory by direct mechanotransduction to the nucleus that can evoke lasting epigenetic modifications at the DNA level, such as histone methylation and acetylation. We conclude by projecting how cell culture mechanics can be modulated to generate MSCs, which epigenetically protected against myofibroblast activation and transport desired regeneration potential to the recipient tissue environment in clinical therapies.

## 1. Introduction

**Figure 1:**
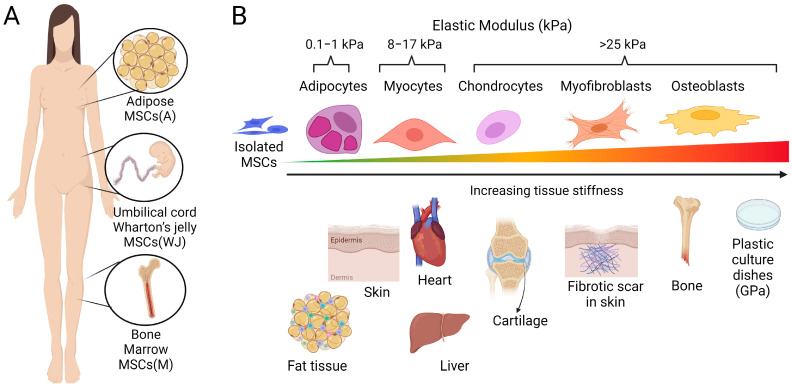
Tissue sources of therapeutic MSCs and stiffness-dependent differentiation. (**A**) The most prominently used tissue sources to isolate therapeutic MSCs from human biopsies include adipose tissues, bone marrow, and umbilical cord Wharton’s jelly. (**B**) The mechanical properties, i.e., softness or stiffness of tissues and those of the culture substrates used to grow and expand adhesive MSCs can influence MSC differentiation capacity and fate. MSCs cultured in soft cell culture environments matched to the elastic modulus (indicated in kPa) of normal fat and muscle tissue exhibit a high propensity for adipogenic and myogenic differentiation. In contrast, growth on stiffer culture substrates promotes the lineage commitment of MSCs towards cartilage and bone. One MSC fate, either representing a transitional state to osteogenesis or an independent scar-forming phenotype, is the activation of MSCs into fibrogenic myofibroblasts. Notably, the scars forming in response to the injury of soft tissues are always stiffer than the normal tissue texture (here schematized for skin), which drives the mechanically induced myofibroblast activation from resident and delivered mesenchymal cells. Cell culture plastic dishes are even stiffer (~10,000-times) than the stiff scar, which results in MSC-to-myofibroblast activation in vitro. Scheme produced with Biorender.

In the late 1960s, Friedenstein and coworkers isolated non-hematopoietic fibroblast-like cells from bone marrow that adhered to culture dishes and formed colonies (clones) in cell culture, with the potential to undergo induced osteogenesis [1,2]. Later, these cells were shown to be able to also differentiate into adipocytes, chondrocytes, and osteoblasts, given the appropriate chemical and mechanical in vitro conditions [3]. Since then, tissue-resident mesenchymal cells with progenitor and multi-lineage differentiation potential have been identified in almost all organs, such as the skin, liver, lungs, kidney, and heart [4]. Based on common defining features, including self-renewal capability, the potential to generate progenitor cells, and differentiation into multiple cell lineages, these multipotent cells are summarized under the umbrella term ‘MSC’, where ‘M’ can stand for ‘multipotent’ or ‘mesenchymal’ and ‘S’ for ‘stem’ or ‘stromal’ [4,5,6,7,8]. MSCs have been isolated for subsequent therapeutic applications from many sources, including but not limited to bone marrow [9], adipose tissue [10,11,12], the perivascular Wharton’s jelly of umbilical cords [13,14], dental pulp tissue [15,16], synovium [17,18], and hair follicles [19]. Throughout this review, we follow the recommendation of the International Society for Cell and Gene Therapy (ISCT) to use the MSC acronym for mesenchymal stromal cells, ‘MSC(M)’ for bone-marrow-derived MSCs, MSC(A) for adipose tissue MSCs, and MSC(WJ) for MSCs from umbilical cord Wharton’s jelly [20] (Figure 1). Because of their regenerative, immunomodulatory, and immune-evasive attributes, both allogenic and autologous MSCs have become invaluable sources for tissue engineering and therapeutic applications aimed at treating a wide range of disease conditions, including the repair of damaged tissues [11,21,22,23,24,25].

When the adult human body is injured, the lost tissue structure is restored by tissue-resident mesenchymal progenitors activated to produce and organize extracellular matrix (ECM) [4,26]. Dysregulated persistent activation of repair cells results in the excessive formation of stiff scar tissue—a condition called fibrosis—affecting all organs [27,28]. We have recently reviewed the roles of tissue-resident mesenchymal progenitors and their fibroblast cousins in physiological and pathological healing [29,30]. In this review, we discuss the fates of therapeutic MSCs that are isolated from tissue biopsies and, after cell culture expansion, delivered to injured tissues with the hope of regenerating excessive or chronic damage that exceeds the body’s own capacity to heal. Notably, delivered MSCs face the same chemical and physical environment that activates tissue-resident mesenchymal cells into so-called myofibroblasts, which are the scar-makers [31]. We propose that balancing beneficial transient and detrimental persistent MSC-to-myofibroblast activation is critical to enhancing the success of MSC therapies.

One pivotal factor contributing to the activation of myofibroblasts is of mechanical nature—specifically, the low deformability or high ‘stiffness’ of their microenvironment. We elaborate on how the mechanical properties of stiff recipient scar tissue and the culture surfaces used to multiply therapeutic MSCs affect their activation into scar-producing myofibroblasts. We discuss the molecular mechanisms of MSC mechanoperception, such as integrins and stretch-activated channels, and MSC mechanotransduction into transcriptional programs via the cytoskeleton and co-transcription factors YAP, TAZ, and MRTF. While acute cellular mechanotransduction is comparably well studied, the molecular mechanisms of mechanically induced permanent cell behavior are only beginning to be understood. Such ‘mechanical memory’ is of high relevance for the permanent MSC-to-myofibroblast activation during cell culture expansion. We explain how prolonged mechanical stress creates persistent myofibroblast memory by direct mechanotransduction to the nucleus, which evokes lasting epigenetic modifications, such as histone methylation and acetylation. We conclude by projecting how cell culture mechanics can be modulated to generate MSCs that are epigenetically protected against myofibroblast activation and transport-desired regeneration potential to the recipient tissue environment in clinical therapies. We restrict our discussion to the cellular aspects of MSC therapies, remaining cognizant of the fact that the mechanical and chemical properties of delivery materials and scaffolds also have a profound impact on MSC survival, performance, and differentiation capacity. For an overview of biomaterials used in the context of MSC therapies, the inclined reader is referred to recent reviews on the subject [32,33,34]. 

## 2. Defining Features of MSCs

Stem cell societies form committees on an annual basis to discuss and standardize the definitions and acronyms of MSCs [20,35]. Given the sensitivity of the issue, we dedicate this first section to a summary of features and markers that define therapeutic MSCs. We pay particular attention to their discrimination from ‘ordinary’ fibroblasts—as much as there is one.

### 2.1. MSC Markers

Many of the now widely used conventional MSC molecular markers were established when MSCs were first discovered and isolated from bone marrow, and later consolidated into a consensus statement [35]. To discriminate and sort MSCs from hematopoietic and vascular cells in the bone marrow by flow cytometry, mesenchymal surface markers are suitable identifiers, such as CD29 (β1 integrin), CD44 (hyaluronan receptor), CD90 (Thy-1), CD73 (ecto-5′-nucleotidase), and CD105 (endoglin-1) (Table 1). In contrast, MSCs do not typically express the endothelial cell junction protein CD31 (also known as PECAM-1) and markers of hematopoietic cells such as CD11b, CD14, CD19, CD34, CD45, CD79α, and CD117 [3,35]. As always, there seem to be exceptions to the rule [36]. Later, MSCs were also described to express human leukocyte antigens (HLA) class A, B, and C (HLA-ABC), but not the HLA-DR isotype, which is expressed on professional immune cells [37,38]. HLAs are key components of the major histocompatibility complex (MHC) class II, which regulates immune cell responses by presenting antigens processed from extracellular proteins [39]. The same surface marker combinations are often also used to identify MSC(A) isolated from fat [40] and MSC(WJ) from umbilical cord tissues [41]. 

However, CD29, CD44, CD90, CD73, and CD105 (but not HLAs) are all expressed by fibroblasts and some are used as fibroblast markers [42]. Therefore, these proteins satisfy the ‘mesenchymal’ criterion but not the possible ‘stem cell’ nature of MSCs. In fact, the lack of true progenitor potential distinguishes fibroblasts from MSCs: while fibroblasts can only imperfectly repair damaged adult tissues, MSCs are considered to have true regenerative potential [31,43]. Thus, to tell MSCs apart from fibroblasts and other mesenchymal cells, additional discriminators are still being sought after. For instance, the mRNA and protein expression analysis of MSC(A) from 15 donors revealed ‘novel’ MSC markers, including CD36, CD271, CD200, CD273, CD274, CD146, CD248, and CD140B, which is the pericyte marker platelet-derived growth factor receptor beta (PDGFRβ) [44] (Table 1). Among these, CD273 and CD146 may indeed be MSC-specific, whereas CD36 (a receptor for thrombospondin-1) [45], CD200 (also expressed on tumor cells) [46], CD274 (PDL-1) [47], and CD248 (endosialin) [48] are also expressed in fibroblasts, often in a tumor context. Arguably, the definition of ‘fibroblast’ provided in the respective works may not always be accurate, and these studies could, in fact, have dealt with MSCs. Because it is difficult to discriminate MSCs, fibroblasts, and perivascular progenitor cells based on molecular markers alone [4], it is essential to also consider functional MSC criteria.

### 2.2. The Regenerative Capacity of MSCs

The ‘regenerative potential’ of MSCs conventionally refers to their capabilities of self-renewal and to differentiate into different cell lineages and thus replenish adult differentiated cells that are lost by tissue wear and tear or injury [6,49]. Self-renewal is the capability of MSCs to divide into daughter cells that preserve the undifferentiated state (stemness) [50,51,52]. Thus, one single MSC can generate colonies of cells with identical characteristics, i.e., they are clonogenic [53,54,55]. When subjected to physical and/or chemical stimuli, MSCs have been shown to differentiate into cells of mesenchymal lineage, including adipocytes [56,57], chondrocytes [58,59,60], osteoblasts [57,61], striated muscle cells [62,63,64,65], vascular smooth muscle cells [66], and possibly even epithelial hepatocytes [67,68,69]. MSC differentiation towards one lineage typically limits the potential to differentiate into another cell type [4,57,70,71]. For instance, adipogenesis and osteogenesis appear to be mutually exclusive fates; MSC subpopulations with high osteogenic potential were shown to exhibit lower adipogenic capacity, and vice versa [72]. We discuss further below how these two ends of the mesenchymal fate spectrum are governed by physical factors, with mechanical stress promoting MSC fibrogenesis and osteogenesis, and a ‘relaxed’ environment favoring adipogenesis [73,74]. 

### 2.3. MSCs Immune Privilege

The grafting efficacy of MSCs after delivery to sites of injury is a major factor determining their therapeutic value, in particular that of allogeneic, i.e., donor-foreign MSCs [37]. Low expression of the co-stimulators of T-cells, CD80 and CD86, and HLA-DR was long believed to allow allogeneic MSCs to escape detection by the recipient’s immune system [41,75]. However, the concept of MSC immune privilege has been challenged by studies showing that allogeneic MSCs can also trigger a host immune response. For instance, allogeneic major MHC molecules expressed by donor MSCs are detected by the T-cell antigen receptor, leading to the expansion of CD4-positive and CD8-positive T-cells [76]. T-cells produce alloantibodies, eventually leading to the rejection of MSCs transplanted in human clinical trials [37,77,78] and different animal models, including mice [76], horses [79], and pigs [80]. The factors regulating immune privilege versus the immunogenicity of MSCs remain unclear. One explanation is heterogeneity among MSCs with respect to their MHC expression [81,82]. Recent studies have also indicated that the immunogenicity of allogeneic MSCs is influenced by the differentiation stage, which determines the expression of surface markers, including MHC [83,84]. These studies suggest that undifferentiated MSCs exhibit lower immunogenicity compared to differentiated MSCs that are detected by the host immune system. Although recipient immune reactions can lead to the rejection of grafted MSCs, there is also therapeutic potential in transplanted MSCs that regulate the host immune system. We discuss the beneficial immunomodulatory actions of MSCs in the later section on their therapeutic application. However, first, we need to consider that not all MSCs are the same—which carries both risks and benefits for MSC therapies.

### 2.4. MSC Heterogeneity

An important consideration for the use of MSC in therapies—and related to the difficulty in defining these enigmatic cells—is their inherent heterogeneity. MSC heterogeneity exists across different donors [85,86,87,88], across different body compartments and tissues [87,89,90,91], and even within MSC populations isolated from the same tissue and donor [6,8,91,92,93]. Of note, MSC features also differ between species, which has implications for the translation of findings made with animal models to potential human applications [94,95]. MSC heterogeneity has historically been demonstrated at the levels of cell phenotype, morphology, and biophysical properties [96]. More recently, epigenetic signatures [92,97], and transcription profiles [72,92,97] have been used to profile MSCs, and much is still to be learned from novel sequencing technologies.

It emerges that chondrogenic, fibrogenic, and osteogenic potentials can differ substantially within one MSC population [98,99,100]. Single-cell RNA-sequencing (scRNA-seq) analysis of freshly isolated human MSC(M) revealed three distinct similarity clusters of MSCs mainly associated with (1) osteogenic markers such as *ALPL* (alkaline phosphatase), *COL1A1* (collagen type 1), and *CD146* (melanoma cell adhesion molecule), (2) adipogenic markers like *ADPQ* (adiponectin) and *MGP* (matrix Gla protein), or (3) markers of chondrogenesis, including *APOD* (apolipoprotein D) (Table 1). Two additional clusters lacked differentiation markers but were enriched in the expression of genes related to self-renewal pathways, such as ribonucleoprotein [101]. Similar clustering for transcriptional similarities was performed with freshly isolated mouse bone marrow, delivering seven sub-populations, one of which was enriched in MSCs [102]. Computational trajectory analysis predicted that cells belonging to the MSC cluster will undergo different degrees of lineage commitment into the other six main clusters: Three of these clusters expressed osteogenic transcription factors such as *Sp7*, *Creb3l3*, *Mef2c*, runt-related transcription factor 2 (*Runx2*, also called CBFα1); two clusters were characterized by the expression of the adipogenic transcription factors *Maff* and peroxisome proliferator-activated receptor gamma (*Pparg*); and one cluster by the expression of various pre-osteoblast and chondrogenic transcription factors [102]. MSC heterogeneity was experimentally corroborated using functional assays for osteogenesis, adipogenesis, chondrogenesis, and clonogenicity after sorting mouse MSC(M) using a hierarchical flow gating strategy after excluding CD31-positive endothelial cells and CD45-positive hematopoietic cells. Subsequent positive sorting criteria included protein products of the main differentially expressed genes for each MSC cluster, such as CD24a, CD31, CD39 (*Entpd1*), CD54 (*Icam1*), CD121b (coding for the interleukin 1 receptor type II), Sca-1 (*Ly6a*), and Ly6c1 (Table 1). The outcome of the functional assays aligned with the differentiation propensity of each specific cluster as predicted by scRNA-seq analysis [92]. Collectively, these studies provide strong evidence for intra-population heterogeneity among MSCs for trilineage differentiation and clonogenicity. It will be a future task to harness this knowledge for specific therapeutic applications. For instance, by selecting and sorting suitable MCS sub-populations for the repair of bone, cartilage, or inflammation and fibrosis.

For such directed strategies to succeed, one needs to consider that MSC heterogeneity is introduced during MSC cell culture, which is pivotal for the large-scale production of therapeutic MSCs [44,103]. Multiple recent scRNAseq studies have been performed with human MSCs subcultured for different numbers of passages. MSC(M) cultured for one passage, separated into several similarity clusters, and divided into three main types of MSCs: (1) ‘Functional’ MSCs, characterized by the expression of the adipokine chemerin and its chemokine-like receptor 1 (CMKLR1), the latter being associated with osteogenic differentiation and immunomodulatory capacity [72]. Enhanced osteogenesis of CMKLR1-positive MSCs was confirmed in vitro and in vivo models, and CMKLR1 immunomodulatory effects were confirmed by the expression of *CCL2*, *TGFB1*, *IGFBP2*, and *PTX3* in culture assays [72]. (2) ‘Self-renewal MSCs’ with high stemness that lacked chemerin and CMKLR1 were characterized by high expression of the stem cell transcription factor *SOX4*, *CD26* (dipeptidyl peptidase-4), and *GAS1* (coding for growth arrest-specific protein 1). (3) ‘Proliferative MSCs’ characterized by the enhanced expression of cell-cycle-related genes [72] (Table 1). 

Another study analyzing human MSC(M) and MSC(WJ) after 6–7 culture passages delivered six distinct scRNA-seq clusters, of which three had transcription profiles and transcription factor predictions related to multi-lineage differentiation capacity [97]. One of these clusters was predicted to have tri-lineage differentiation potential into chondrocytes based on the expression of *TRPS1*, *SCX*, *COL11A1*, osteoblasts (*JUN*, *ATF4*, *ID4*), and adipocytes (*CEBPB*, *PPARG*). The second cluster displayed gene expression signatures associated with both chondrocytes and osteoblasts (*OMD*, *ASPN*, *GPM6B*, *IFITM1*, and *GPNMB*), and the third showed adipogenic specialization (*EBF2* and *HMGA2*) [97]. The fourth ‘stemness’ cluster was characterized by the expression of proliferation markers such as *TOP2A*, *MKI67*, *E2F1*, and *CCNA2*, as well as pluripotency and self-renewal markers including *E2F8*, *CTCF*, *PBX3*, and *MYBL2*. The fifth cluster was enriched in genes related to immunomodulation, such as *CD106* (VCAM1), which mediates leukocyte adhesion, *CD47* serving as a ‘don’t eat me signal’, *CD248* coding for part of the T-cell receptor, and the receptor for urokinase plasminogen activator *CD87* (Table 1). The sixth cluster comprised a small population expressing genes associated with a smooth muscle and/or myofibroblast phenotype, including α-smooth muscle actin (α-SMA) (*ACTA2*), the myosin light chain *MYL6*, and the tropomyosin *TPM2* [97]. Another scRNAseq study performed with MSC(WJ) in the first culture passage clustered into five distinct populations that were characterized by differences in the expression of genes associated with collagen, proliferation, chemokine production, and aging [104]. Common to all MSC subclusters produced by these RNA-seq studies were transcriptome signatures indicating differentiation capacity, self-renewal, and immunomodulation. This commonality is probably less surprising considering that these very features were used to annotate ‘MSCs’ in the first place. Nevertheless, scRNA-seq profiling and upcoming meta-analysis across different datasets hold great future promise for identifying MSC populations with desired properties for therapeutic purposes.

Further below, we consider how the mechanical environment of conventional cell culture conditions affects the quality and homogeneity of therapeutic MSCs, as well as myofibroblast activation [103,105,106,107,108]. In addition, MSC heterogeneity is introduced by different culture conditions and methods, such as oxygen levels, glucose levels, growth factor supplements, and the choice of fetal bovine serum or human platelet lysate used as cell culture additives (reviewed in [109,110,111]). In-depth discussion of decades of MSC cell culture condition refinement would exceed the scope of this review; it suffices to say that standardizing culture conditions is critical to minimize MSC variability and ensure consistent MSC quality in good manufacturing practice conditions for clinical applications.

## 3. Benefits and Risks of Myofibroblast Activation in MSC Therapies—An Example of Skin Wound Healing

Therapeutic MSCs have three main fields of application: (1) accelerating and supporting the repair of severely or chronically damaged tissues [112,113]; (2) exerting control over the immune system to allow tissue regeneration [114,115,116]; and (3) managing autoimmune diseases [117,118,119]. To maintain focus, we use the healing of severe skin wounds, such as those created by burn injuries, as a paradigm application for therapeutic MSCs that can be jeopardized by MSC-to-myofibroblast activation [120,121,122]. Other skin applications of therapeutic MSCs include the support of chronic non-healing wounds such as diabetic foot ulcers, delivery to reduce scar formation and fibrosis, and aesthetic applications such as skin rejuvenation. For these applications, the reader is referred to other reviews [123,124,125]. Notably, the fundamental mechanisms of successful and dysregulated tissue repair by endogenous and exogenous mesenchymal cells, including MSCs, are conserved across all organs.

The capacity of our body to heal fails in cases of substantial damage, such as burn injuries or severe trauma. Large area burn wounds are a leading cause of morbidity and impose a significant burden on global health care costs [126,127,128]. The 2016 American Burn Association Report states that survival rates of burn victims decrease below 50% when the area of damaged skin exceeds 65% of the total body surface area [129]. Surviving patients spent approximately one day in hospital for each percent of burned area, and with >10% of area burnt, the average treatment costs are $257,582 [129]. Even if patients survive, the dysregulated healing of large area burns causes dramatic contractures [130]. Out of 1865 burn patients, 620 developed post-burn hypertrophic scars in the US in 2015 [131]. Fibrotic scarring has an enormous impact on the patient’s quality of life due to functional skin limitations and poor aesthetics [132]. Depression and anxiety are common among burn patients [133]. In patients with severe burns, infection-related sepsis accounts for >75% of all deaths [134]. Covering debrided wound surfaces with surgical interventions reduces infection risks and enhances patient survival [135,136]. The standard of care consists of covering burn wounds with meshed skin allografts or autografts produced from uninjured body regions. Because of the limited availability of skin grafts, different skin substitutes are used in the clinic: (1) cell-free polymer scaffolds; (2) cell-laden scaffolds; (3) therapeutic cells alone; and (4) self-assembled skin equivalents with multiple cell types [126,136,137,138,139,140]. Because autologous skin fibroblast donor sites are scarce after large area burns and allogenic fibroblasts bear the risk of rejection, MSCs are intensively explored for therapeutic skin wound healing applications [141,142,143,144,145,146,147]. MSCs isolated from different sources have entered pre-clinical and clinical trials to treat skin wounds, including MSC(M), MSC(WJ), MSC(A), and MSCs derived from gingiva, and burn eschar [145,148,149,150,151,152] (Table 2). In all applications, MSCs are key in forming a mature granulation tissue that supports epithelialization and vascularization either by producing and remodeling wound ECM or secreting factors that orchestrate the actions of other cells in the wound environment [128,141,153,154]. 

### 3.1. The Immunomodulatory Actions of Therapeutic MSCs—The Key to Scarless Healing?

One advantage of using MSCs in wound healing therapies is that in some cases they seem to promote the regeneration of skin appendages, angiogenesis, and vascular stability without allowing fibrosis to occur [155,156]. The fibrosis-suppressing nature of MSCs has been attributed to their proposed role as ‘rheostats’ that sense the healing environment and accordingly produce factors that keep pro-fibrotic immune cells at bay [156]. MSCs communicate with cells of the host immune system either through direct contact as discussed in the above section on immune privilege and/or through secreted factors such as extracellular vesicles, chemokines, cytokines, and growth factors [38,157,158,159]. For instance, MSC secretion of prostaglandin E2 (PEG2) was shown to regulate the proliferation and balance of T-cell subtypes [160] and the maturation and antibody production of B-cells [117]. By secreting interleukin (IL)-10, arginase-1, tumor necrosis factor-α (TNF-α) and PEG2, MSCs also instruct macrophages to acquire anti-inflammatory phenotypes that can support scar-less healing of skin injuries [117,160,161]. Further repair-promoting factors secreted by MSCs include TNF-α-stimulated protein 6 (TSG-6) [157], hepatocyte growth factor (HGF) [156,160], vascular endothelial growth factors (VEGFs), basic fibroblast growth factor (bFGF), and TGF-β1 [160]. 

However, there is also a risk that the diseased host environment changes the immunomodulatory and other wound healing features of the delivered MSC and thereby worsens the condition rather than improving it. For instance, the inflammatory milieu present in autoimmune diseases was shown to turn therapeutic MSCs with intended immunosuppressive actions into immunostimulatory cells that exacerbated inflammation [158,162,163,164]. This vulnerability can be alleviated by priming therapeutic MSC during the culture expansion phase, i.e., preparing them to fulfill a specific function. MSC priming for better wound healing outcomes can be achieved by genetic manipulation, e.g., to overproduce HGF and IL-10 [165], or culture treatment with cytokines, hypoxia, and pharmacological agents [166,167,168]. In a section further below, we discuss the novel concept of mechanical priming.

An alternative strategy to mitigate the risk of MSCs being converted into undesired agents by host(ile) environment is the delivery only of the MSC secretomes—or fractions thereof (Table 2). For instance, exosomes were found to transport the immunoregulatory, regeneration, and wound-healing properties of MSCs in clinical settings—without the need for MSCs to be present [38,169,170,171,172,173]. Exosomes belong to the group of secreted extracellular vesicles, which also includes ectosomes, microvesicles, and apoptotic bodies [38,174]. According to the guidelines of the International Society for Extracellular Vesicles, exosomes are <200 nm in diameter and derive from endosomes, whereas microvesicles are >200 nm and are formed by plasma membrane pinching; both types transport cytokines, microRNA (miR), and mRNA to neighboring cells [175]. Treatment with MSC(M)-derived exosomes skewed mouse macrophages towards anti-inflammatory polarization states in vitro and in mouse wounds via delivery of miR-223 that targets the macrophage polarization regulator *Pknox1* [176]. Scarring of mouse wounds is also suppressed by the delivery of TSG-6 via exosomes from MSC(M), leading to a reduction in the inflammatory factors monocyte chemoattractant protein-1 (MCP-1), TNF-α, IL-1β, and IL-6 in the wound bed [177]. Likewise, the presence of miR-21, miR-146a, and miR-181 and miR-181c in exosomes from MSC(WJ) results in higher numbers of anti-inflammatory macrophages [178] and suppression of inflammatory macrophages through toll-like receptor 4 (TLR-4) and NF-κB inhibition in rodent wounds [179]. MSC-derived exosomes also ameliorate healing by positively regulating other cells in wounded skin, such as epithelial and endothelial cells [180]. Delivery of Wnt4 protein with MSC(WJ)-derived exosomes promotes closure and re-epithelialization while inhibiting cell apoptosis in skin burn injury animal models [181]. MSC exosome delivery of angiopoietin-2 [182], miR-31 [183], and early growth response-1 (EGR-1) [184] all induce endothelial cell proliferation and angiogenesis, which accelerate the healing process.

Major pro-healing and anti-scarring actions of MSC-derived exosomes have also been attributed to their actions on wound fibroblasts. MSC(A)-derived exosomes were shown to reduce skin fibrosis in an animal model of systemic sclerosis by supplying miR-29a-3p, which decreases the expression of the anti-apoptotic genes Bcl2 and Bcl-xl, and by suppressing the expression of pro-fibrotic PDGFRβ, although this study did not experimentally associate the effects with specific skin cell populations [185]. MSC(WJ) exosomes enriched with miR-21, miR-23a, miR-125b, and miR-145 delivered to skin wounds of animal models, accelerate healing and reduce fibrosis by targeting and thereby reducing the expression of SMAD2, a key mediator downstream of pro-fibrotic TGF-β signaling [186]. MSC(WJ) exosomes are also rich in miR-21-5p and miR-125b-5p which are predicted to target and reduce the expression of TGF-β receptor types I and II and, thus, TGF-β1 signaling in animal skin wound fibroblasts [187]. Likewise, delivery of TSG-6 with MSC(M) exosomes to mouse skin wounds results in reduced phosphorylation of SMAD2/3 in fibroblasts [177]. The overall outcome of reduced TGF-β1 signaling in fibroblasts is the suppression of a pro-fibrotic phenotype commonly known as the myofibroblast.

### 3.2. The Benefits and Risks of MSC-to-Myofibroblast Activation

One function of therapeutic MSCs that cannot be achieved by delivering their secretome alone is the production of collagen-rich granulation tissue [188,189]. The reconstitution of lost ECM is particularly important to support the healing of large area wounds and severely damaged tissues. The combination of collagen ECM production and its contraction into mechanically stable scar tissue was name-giving for the myofibroblast [29,30], a cell activation state originally described for wound granulation tissue fibroblasts [190]. Other tissue-resident mesenchymal myofibroblast precursors, in addition to those summarized under the term ‘fibroblast’ [191,192,193], include adipocytes, pericytes, smooth muscle cells, and local MSCs (reviewed in [4,194,195,196]). 

Myofibroblast activation from all these different precursors, including delivered therapeutic MSCs, is now understood to follow a multi-step process [31]. Stimuli arising during tissue damage like inflammation and changes in ECM architecture activate fibroblastic cells to proliferate, migrate, and produce collagen. With increasing mechanical resistance of the developing wound granulation tissue, activated fibroblastic cells increasingly form stress fibers, which are contractile bundles constructed of filamentous actin and non-muscle myosin [197]. Initially, stress fibers contain β- and γ-cytoplasmic actins; the corresponding cell activation state is often called ‘proto’-myofibroblast [198]. Subsequent myofibroblast activation stages are defined by de novo expression of α-SMA; incorporation of α-SMA into stress fibers confers higher contractile activity compared to equivalent amounts of the cytoplasmic actins [199]. Myofibroblast contractile activity promotes wound closure of injured skin and organizes ECM into mechanically resistant scar tissue in other organs; the depletion of α-SMA or pharmaceutic inhibition disrupts the normal process of wound healing [200,201]. Although expression of α-SMA is frequently used to discriminate myofibroblasts from their non-contractile precursors, it is not an exclusive marker [105]. Smooth muscle cells and pericytes in the wound environment also express α-SMA, albeit in different organizations, i.e., not in stress fibers [202]. For a more extensive discussion of myofibroblast activation states and their markers, we refer to our recent reviews on that topic [29,30,31]. Physiological tissue repair ideally terminates when the lost ECM has been restored and inflammatory cells and myofibroblasts are gradually cleared by programmed cell death, i.e., apoptosis [203]. However, dysregulation of myofibroblast behavior and their persistent activity leads to pathological accumulation of ECM and remodeling of ECM fibers. The resulting augmented tissue stiffness establishes a feedback loop that sustains myofibroblast activation and can ultimately result in the severe hypertrophic scarring that characterizes fibrotic tissues [200,203,204,205,206]. 

Since our body generates myofibroblasts from all available sources to rapidly repair injuries, the wound environment of severely injured skin—in fact, of all damaged organ tissues—will also convert engrafting therapeutic MSCs [26,27,42,207,208,209,210]. Myofibroblast activation of delivered MSCs per se can be a wanted effect to enhance the healing process, but it also bears the risk of severe scarring if myofibroblastic MSCs do not cease their actions. For instance, excessive myofibroblast activation of delivered MSCs contributes to skin scarring and contraction of MSC-populated scaffolds used for skin tissue engineering [42,211,212,213]. The progression of healing and the presence of a fibrotic scar environment at the time of MSC delivery seem critical. MSCs engrafted into early scar stages were shown to improve organ healing; MSCs delivered to mature scars are prone to fibrogenesis in the fibrotic skin, lung, kidney, liver, and fibrotic heart [42,214,215,216,217,218,219,220,221,222,223]. Even before delivering therapeutic MSCs to damaged tissues, they are at risk of turning fibrogenic during the culture expansion process on typically stiff adhesive surfaces. We next discuss how mechanical stimuli control MSC fates by focusing on the stiffness or softness of their substrate. In the following section, we explore how the mechanosensitivity of MSCs can be used for standardized and large-scale production of resilient MSCs capable of thriving in harsh wound environments.

## 4. Mechanically Driven MSC Fates—Acute Mechanosensing and Mechanical Memory

Physical cues that tissue-resident or delivered MSCs experience by adhering to a substrate involve tissue strain [224], porosity [225], dimensional variations, surface patterns, as well as hydrodynamic shear stresses, and forces applied directly from neighboring cells [226,227,228,229,230]. A key mechanical factor affecting MSC lineage differentiation and myofibroblast activation is the stiffness of their ECM substrate. Biologists typically say ‘stiffness’ or ‘rigidity’ when they mean how deformable a material or tissue is, i.e., how much force per unit area (stress) is required to induce a length change (strain) in the material. Physicists refer to stress over strain as Young’s modulus, *E*, for elastic materials, with unit Pascal (Pa) [231,232]. Notably, tissues are not perfectly elastic, and, in fact, strain stiffening is an important characteristic of biological materials [233]. Nevertheless, tissues behave approximately elastic upon small deformations at the single cell level (a few microns), and the Young’s modulus in these cases provides an appropriate measure [234]. Tissue stiffness hinges on both the composition and organization of the ECM [235,236]. Fat tissue, skin, and the brain are examples of softer tissues (≈0.5–4 kPa), while bone represents the most rigid tissue in our body (~15,000–20,000 kPa); muscle and cartilage fall between these ranges, with ~10–50 kPa and 1000 kPa, respectively [234,237,238] (Figure 1). With the exception of cartilage and bone, and repair or fibrotic scar is always stiffer than the tissue of the organ where it forms (~50–100 kPa) [231,237]. For instance, the elastic modulus of fresh, healthy human skin has been measured with atomic force microscopy to be in the range of 0.5–10 kPa [239]. As the skin heals after injury, the provisional fibrin ECM becomes gradually replaced by collagen, and wound stiffness increases with ongoing collagen remodeling to reach moduli of ~20 kPa [240]. Transient stiffening is required to protect wound tissue from rupture, but the dysregulated healing of untreated large area wounds inevitably results in scar contractures. The ECM of hypertrophic scars reaches 50–100 kPa, i.e., 100 times stiffer than normal skin [241,242,243,244]. The functional consequences of the fibrotic scar being stiffer than the host tissue are wide ranging and severe. In addition to destroying organ function, the stiff fibrotic scar drives the progression of fibrosis by turning various healthy precursor cells into fibrotic myofibroblasts [245,246,247]. For instance, in healing rat skin wounds, expression of α-SMA is accelerated along with enhanced tissue tension by preventing wound contraction with plastic frames [248,249]. Likewise, stretching human burn scar tissue in situ enhances fibrogenic features [250]. 

To elucidate how MSCs respond to mechanical forces, various culture devices have been used, often reducing the system to one specific mechanical cue [125,251,252,253,254]. Because MSC cell manufacturing is typically performed on planar surfaces such as plastic culture flasks or plastic beads in bioreactors [55,255], we focus here on the discussion of how to manipulate MSC fate mechanically on two-dimensional surfaces by modulating substrate stiffness. Exploring how physical cues affect MSC behavior and fate in three-dimensional culture constructs and scaffolds is also critical for tissue engineering application, as discussed elsewhere [256,257,258]. To replicate physiological and pathological stiffness conditions in vitro in 2D, stiffness-tunable proteins or synthetic hydrogels, polyurethanes, and silicones are the most widely used materials [259,260] (Figure 1). 

Mechanical cues were shown to impact MSC immunomodulatory behavior through the NF-κB pathway signaling, influencing how MSCs can recruit immune cells to damaged tissues [261,262,263]. Seminal experiments performed with MSCs cultured on differently stiff substrates showed that an elastic modulus of 8–17 kPa favors myogenic fate choice (MyoD expression), while culture on 25–40 kPa substrates matching the stiffness of pre-bone osteoid ECM promoted osteogenic commitment, as shown by increased expression of RUNX2 [73]. Induced osteogenesis of MSCs on stiff culture substrates generally comes at the expense of adipogenesis—and vice versa [125,264]. It appears that myofibroblast activation of MSCs—like that of fibroblasts—can occur at substrate stiffness above ~15 kPa in standard medium. Rather than being a lineage fate, myofibroblast activation may be considered an intermediate step on the path to osteogenesis, in which MSCs will remain if chemical osteoinductive factors are missing [251]. The same study showed that mechanical myofibroblast activation of MSCs also reduces regenerative features and their capacity to undergo adipogenesis in a process that involves the mechanoresponsive transcription factor Yes-associated protein (YAP). In the next section, we provide an overview of how adherent cells, including MSCs, sense the mechanical environment and transduce the physical signal into chemical and transcriptional signaling responses.

### 4.1. Mechanoperception Mechanisms of MSCs—And Other Adherent Cells

**Figure 2:**
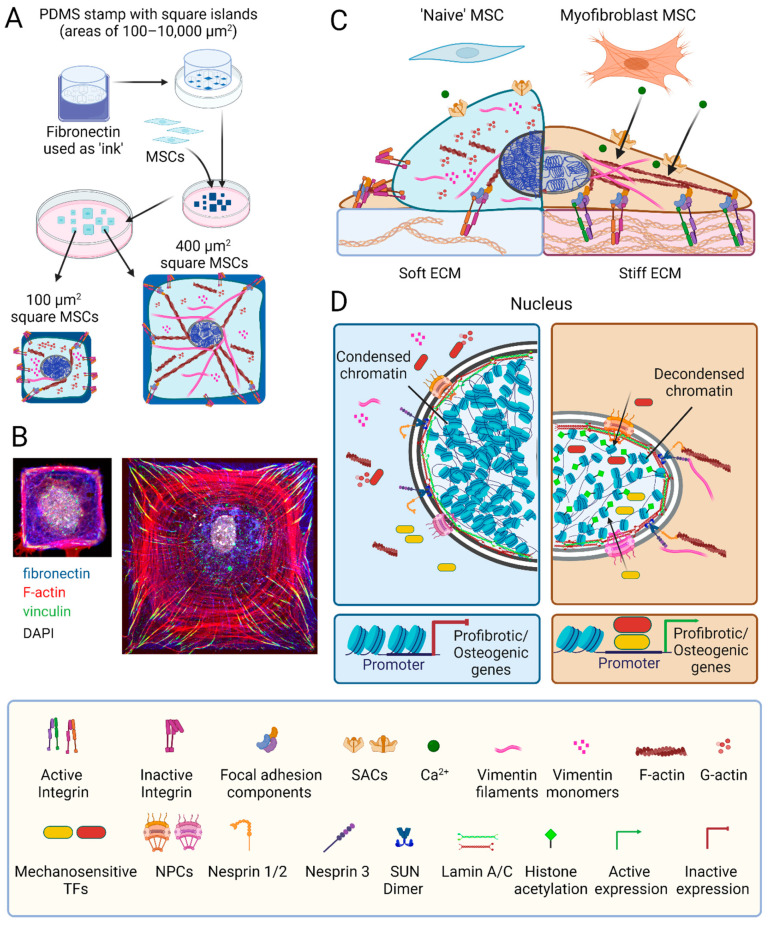
MSC mechanoperception and nuclear mechanics. (**A**) The spreading area of mesenchymal stromal cells (MSCs) attaching to an adhesive substrate can be controlled using micropatterning; for instance, by transferring fibronectin protein (blue staining) in square shapes of different areas onto glass or plastic substrates using polydimethylsiloxane (PDMS) stamps. (**B**) Restricting MSC spreading limits the number and size of focal adhesions (green vinculin staining) and F-actin stress fibers (phalloidin, red), thus overall reducing MSC stress. (**C**) Another way to reduce stress on MSCs in culture is manipulating the elastic modulus of their substrate. MSCs perceive mechanical cues from the extracellular matrix (ECM) via transmembrane integrins; binding to extracellular ligands and intracellular F-actin shifts integrins from a low affinity inactive to a high-affinity active configuration. This integrin conformational switch prompts the assembly of complex focal adhesion structures comprising the cytosolic proteins talin, vinculin, focal adhesion kinase, paxillin, and filamin. Focal adhesions serve as hubs for mechanotransduction pathways, orchestrating the polymerization of G- into F-actin and the organization of vimentin monomers into intermediate filaments. Mechanical stress also opens stretch-activated channels (SACs) to allow the influx of Ca^2+^ into the cytosol to trigger distinct signaling cascades. (**D**) The nuclei of MSCs grown on stiff surfaces are characterized by higher lamin A:C ratios in the inner nuclear membrane, more decondensed chromatin and higher histone acetylation compared to soft environments. A direct connection between ECM adhesions and the nucleus is established through the nucleoskeleton and cytoskeleton complex (LINC), containing nuclear envelope spectrin repeat proteins (nesprins) and Sad1p and UNC-84 homology (SUN) proteins that span the nuclear envelope. Nesprin-3 attaches SUN proteins to F-actin, whereas nesprins-1 and -2 link to intermediate filaments. Within the inner nuclear membrane, SUN dimers interact with lamin A bound to chromatin, causing organized DNA to unfold under high mechanical stress. High stress enhances the nuclear translocation of mechanosensitive transcription factors, such as MRTF-A, Runt-related transcription factor 2 (RUNX2), Yes-associated protein (YAP), and transcriptional coactivator with PDZ-binding motif (TAZ) via opening of the nuclear pore complex (NPC). The promoter binding of these transcription factors drives the expression of pro-fibrotic and osteogenic genes. Scheme elements produced using Biorender, immunofluorescence images produced by Nicole Berezyuk (Hinzlab).

MSCs mainly, but not exclusively, perceive mechanical stimuli from the ECM via transmembrane adhesion receptors called integrins. There are 18 α and 8 β integrin subunits that combine to form 24 different αβ integrins. The different αβ combinations determine the binding specificity of the integrin heterodimer and activate different signaling pathways [265,266]. Application of extracellular or intracellular force results in integrin clustering and recruitment of mechanosensory cytoplasmic proteins, including talins, kindlins, and vinculin; signaling molecules like focal adhesion kinase (FAK), adapter proteins like paxillin; and actin linker proteins like filamin A. Collectively, these components first form so-called focal complexes that transition into mature focal adhesions in a force-dependent maturation process. Focal adhesions not only receive but also transmit forces from the cytoskeleton to extracellular ligands [266]. Focal adhesions allow MSC adhesion and spreading, and play crucial roles in determining stress-dependent MSC differentiation [267]. For example, MSC(M) cultured on 0.1–1 kPa soft substrates reduce the expression of β1 integrins on their surfaces by internalization through caveolae-dependent endocytosis within 2 h [268]. Inhibiting β1 integrin internalization reduces MSC neurogenic differentiation capacity—a lineage commitment that is made on very soft substrates, like the brain. Decreased neurogenesis has been attributed to reduced activation of the RUNX2/Smad/bone morphogenetic protein (BMP) signaling pathway; in contrast, MSCs cultured on stiff substrates exhibit higher surface expression and ECM engagement of β1 integrins, which promote osteogenic differentiation [268,269]. Elegant seminal studies established a correlation between the size of cell adhesion areas and the inclination of MSCs towards either osteogenesis or adipogenesis [74]. Restriction of adhesion area for MSCs using micro-patterning techniques reduces the stress experienced by MSCs on stiff surfaces, favoring adipogenesis and maintenance of stemness features, while increasing adhesion area promotes osteogenesis [270,271,272] (Figure 2). 

Other critical mechanosensitive elements on the cell surface are stretch activated channels (SACs) that are activated by increased membrane tension to allow the passage of cations [273,274,275] (Figure 2). For instance, high substrate stiffness and large adhesion surfaces were shown to increase the frequency of spontaneous calcium oscillations in fibroblasts compared to soft substrates and cell growth on small adhesive islands [276]. Physical cues may also activate SACs to allow calcium influx in MSCs; as a major second messenger, calcium can impact MSC fates through the initiation of various downstream signaling pathways [277,278,279]. SACs, including Piezo channels and some transient receptor potential (TRP) channels, are abundant in MSCs [277,278,280]. Activation of Piezo1 in human MSC(M) promotes osteogenesis while suppressing adipogenesis; this intracellular calcium-dependent effect is mediated through ERK1/2 and p38 MAPK signaling, ultimately enhancing expression of BMP2 [281,282]. Moreover, shear forces stimulate the activation of TRPM7 [283] and TRPV4 [284], initiating calcium-dependent mechanosensitive pathways that result in the increased expression of RUNX2 and enhanced osteogenesis [283,284]. In aging MSCs, Piezo1 levels are reduced, and activation of Piezo1 using the agonist Yoda1 has the potential to enhance MSC function and reduce senescence [281]. Whether and how SACs are involved in guiding MSC fate on differently stiff substrates remains to be shown.

Adherent cells match extracellular resistance with internal contractile stress. Consequently, manipulating cytoskeletal stress by inhibiting myosin action and/or the formation of contractile stress fibers has profound effects on MSC lineage choice [73,251,285,286]. Our own work has shown that the subset of α-SMA stress fiber-positive and highly contractile human MSC(M) is osteogenic with low clonogenicity potential. Knock-down of α-SMA in these cells enhances their adipogenic potential, while overexpression of α-SMA in α-SMA-negative MSCs reduces their adipogenic and clonogenic capacities [251]. In a nutshell, reducing MSC contractility on stiff substrates achieves a similar osteogenic-to-adipogenic switch observed after relaxing MSCs on soft substrates. In all the above studies, MSC lineage decisions were assessed by measuring the levels of transcripts and/or protein products characteristic of the respective lineage. But how does mechanical stress change transcription programs that run in the nucleus?

### 4.2. MSC Mechanotransduction: Mechanosensitive Transcription Factors and the Nucleus

The most widely studied mechanisms through which mechanical stress enhances the transcription of pro-fibrotic and/or pro-osteogenic genes in MSCs are promoting the translocation of the co-transcription factors YAP, transcriptional coactivator with PDZ-binding motif (TAZ), myocardin-related transcription factor A (MRTF-A) [287,288], and RUNX2 [254] from the cytosol into the nucleus. YAP and MRTF-A mediate the expression of profibrotic genes and epigenetic modifiers [289], and RUNX2 regulates the expression of genes associated with MSC osteogenesis [254]. Increased F-actin polymerization and enhanced cell contractility underlie the mechanisms regulating YAP and MRTF-A nuclear translocation. Binding of MRTF-A to non-polymerized globular actin prevents its nuclear import, while the assembly of globular actin into filamentous actin releases the block and liberates MRTF-A for nuclear shuttling [289]. Likewise, incorporation of α-SMA into stress fibers enhances the translocation of YAP and TAZ into the nucleus, which supports MSC osteogenesis [251]. The molecular mechanisms of stress-mediated YAP and TAZ nuclear shuttling are less clear and also involve changes in the nuclear envelope [290]. Applying force to the nucleus results in conformational changes and increases the permeability of the nuclear pore complex for larger proteins; this stress-induced change is sufficient to trigger the nuclear translocation of YAP [291]. 

The nucleus is often the final destination of mechanical cues transmitted from transmembrane integrins in ECM adhesions through the cytoskeletal machinery [287,288] (Figure 2). Mechanical stress enhances the polymerization of cytoskeletal actin and vimentin filaments, which directly transmit mechanical signals from the ECM via integrins to protein complexes that span the outer and inner nuclear envelopes [292,293]. A crucial player in transmitting mechanical signals from the outer to the inner nuclear membrane is the linker of the nucleoskeleton and cytoskeleton (LINC) complex, aptly named for its function. Spanning the nuclear envelope, the LINC complex consists of nuclear envelope spectrin repeat proteins (nesprins), Sad1p and UNC-84 homology (SUN) domain proteins, and Klarsicht, ANC-1, and Syne homology (KASH) domain proteins (Figure 2) [294,295]. Disrupting the LINC complex can change the fate of MSCs cultured on stiff substrates by decoupling the nucleus and cytoskeleton; such MSCs behave like MSCs on soft substrates [254] (Figure 2). This effect is achieved because the LINC connects physically to lamins in the inner nuclear membrane through SUN domain proteins. Mammalian cells express three isoforms lamin A, B, and C, which are prototypical intermediate filaments that form a fibrous meshwork—the nuclear lamina. The ratio between the lamin isoforms changes in response to physical cues and during cell differentiation, including MSC-to-myofibroblast activation [296,297]. Absence of lamin A/C reduces the adipogenic capacity of MSCs, indicating the lamin structures are required for receiving physical cues to change MSC fate [298]. In the context of fibrosis, mutations in lamin A in mice were shown to adversely affect the function of mechanosensitive transcription factors and thereby disrupt the expression of pro-fibrotic genes that contribute, for instance, to cardiac fibrosis [299]. The inner nuclear lamina functions as a mechanoresponsive component of the nucleus by transmitting physical signals directly to chromatin through various protein binding partners, such as LEM (LAP2, emerin, and MAN1), which contribute to the modulation of gene transcription [300]. 

Through this link, the chromatin of MSCs grown on stiff substrates has been shown to undergo global remodeling [301,302]. For instance, the nuclei of MSCs grown in a soft environment contain more compact chromatin, while the nuclei of MSCs on stiff substrate have less condensed chromatin [297,301,303]. Alterations in global chromatin condensation can directly impact gene expression regulatory regions; locally stretching chromatin at specific loci was shown to result in the upregulation of nearby gene regions as fast as within 2 min [304]. Furthermore, transposase-accessible chromatin sequencing (ATAC) studies revealed that the chromatin accessibility of mammary epithelial cells changes in response to physical cues [305]. Hepatic stellate cells exhibit distinct patterns of chromatin accessibility and selective binding of specific transcription factors when grown on soft (1 kPa) versus stiff culture substrates (25 kPa) [306]. Likewise, heart valve fibroblasts exhibit more accessible gene regions on stiff surfaces than on soft surfaces—stress-regulated chromatin openness is related to myofibroblast activation [303]. Another aspect of chromatin remodeling in response to physical cues is epigenetic modification. Less condensed chromatin of MSC(M) in a stiff environment is characterized by higher histone acetylation, possibly mediated by a low expression levels of histone de-acetylase (HDAC) and high levels of histone acetyltransferase (HAT) [301]. Physical cues also alter histone methylation with elevated global histone methylation, reported for human and mouse lung fibroblasts after exposure to stiff environments [307]. In addition to acetylation and methylation of histones, physical stimuli were also shown to induce epigenetic modifications of the DNA. Culture on stiff substrates is associated with high overall DNA methylation in human lung fibroblasts and low global chromatin condensation [308]. Consistently, the protein expression levels of DNA methyl transferases (DNMT) are high in vascular smooth muscle cells cultured on stiff substrates [309]. However, the precise mechanisms through which mechanical stress regulates epigenetic processes in MSCs and how these modifications influence MSC differentiation and/or myofibroblast activation still remain at large.

### 4.3. Mechanical Priming of MSCs for Therapeutic Applications

**Figure 3:**
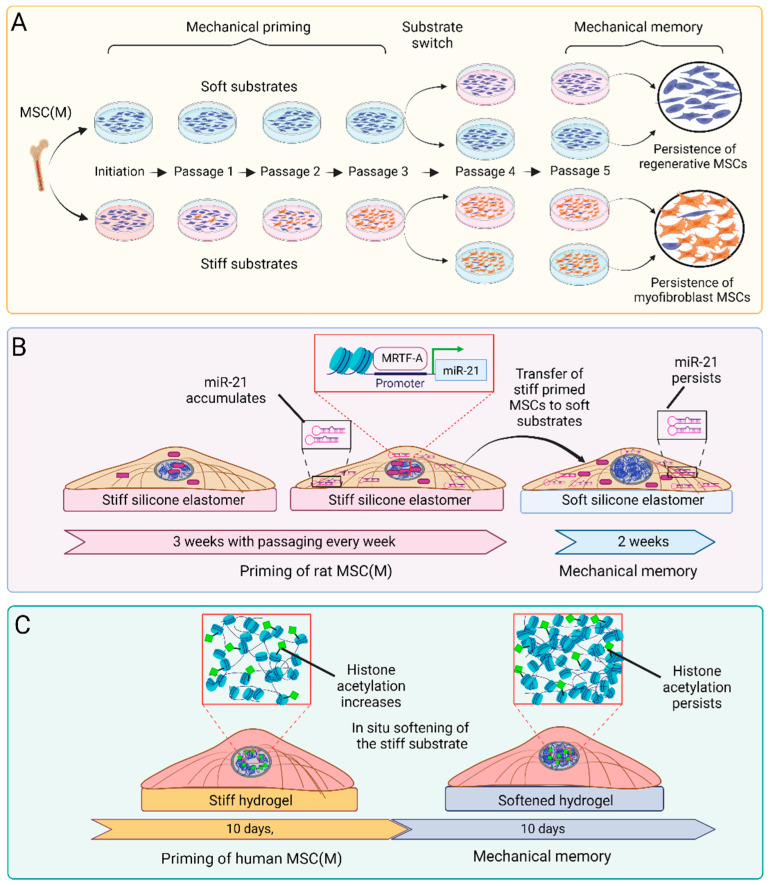
In vitro systems and mechanisms to generate mechanical memory in MSCs. (**A**) Seminal studies generated ‘long-term’ mechanical memory of lung fibroblasts [310] and MSC(M) [125] by culturing and adapting (‘priming’) cells for up to 3 weeks on either soft or stiff silicone elastomer substrates. Mechanical memory was defined as the capacity of MSCs to retain regenerative (soft) or pro-fibrotic and/or pro-osteogenic (stiff) features after switching to the respective substrate for another 2 weeks. (**B**) In the same study, growth on stiff culture substrates was shown to induce nuclear translocation of the mechanosensitive co-transcription factor myocardin-related transcription factor A (MRTF-A), where it drives the transcription of the profibrotic microRNA miR-21 [125]. Cytoplasmic miR-21 levels remain elevated for up to 2 weeks even after switching to soft substrates, whereas MRTF-A relocates to the cytosol within minutes. (**C**) In a different experimental approach to generate ‘short-term’ mechanical memory, MSCs and fibroblasts were cultured on stiff phototunable hydrogels for 10 d to acquire high levels of histone acetylation and low condensed chromatin [301,303]. Following in situ softening of the hydrogels using a light reaction, MSCs maintained high histone acetylation levels while showing increased chromatin condensation. The preserved histone acetylation can regulate chromatin accessibility and transcription profiles.

So far, we have presented how mechanical stress caused by adhesion to stiff substrates controls MSC fate, either acutely or lasting, through epigenetic alterations. We close this review with some considerations on how this knowledge can be exploited to produce therapeutic MSCs that retain mechanically induced features even after delivery to injured tissues. In our own studies, we discovered that the prolonged culture (‘priming’) of fibroblasts on soft (5 kPa) or stiff (100 kPa) silicone polymer surfaces persistently suppresses or enhances fibrotic cell traits that are preserved even after switching to the respective other mechanical condition [310]. We coined this phenomenon ‘mechanical memory’, where ‘prolonged’ in the context of the seminal study means three passages of 1 week each and ‘persistent’ means at least another two passages of 1 week following the substrate switch (Figure 3). Mechanical memory was first studied with rat lung fibroblasts and later confirmed in several studies with different experimental systems and various mesenchymal cell types, including rodent and human MSCs [125,301,310,311,312]. In our own studies, we found it critical that mesenchymal cells are never exposed to GPa-stiff culture plastic surfaces for memory to form, and always directly explant the primary cell isolate onto the respective soft and stiff polymer surfaces [106,125,310]. The most common readout for mechanical memory in these cells is the maintenance of stiff-substrate-acquired myofibroblast phenotype traits even after the switch to soft surfaces that are typically not permissive to induce pro-fibrotic and contractile cell traits, such as the formation of stress fibers and the expression of α-SMA and ECM proteins.

There is little consistency across the different in vitro mechanical memory studies concerning the stiffness-tuneable material, the stiffness values, and the timelines used to achieve priming and test memory, even if published by the same group. For the most part, the differences are due to practical considerations and the systems preferred by the respective laboratories. For instance, 10 day culture on photo-tuneable 36 kPa allyl sulfide hydrogels generates ‘stiff’ mechanical memory in human MSC(M), which is maintained for another 10 days after softening the gels to 5.5 kPa [301] (Figure 3). Human MSC(A) primed for two weeks on 1 kPa soft polyacrylamide gels maintain their soft-primed phenotype and delay the development of pro-fibrotic characteristics for up to one week even when switched to 120 kPa stiff substrates [311]. Pig aortic heart valve fibroblasts acquired myofibroblast features over 7 days of culture on 4 kPa polyethylene glycol hydrogels and memorized these features for 2 days after in situ hydrogel softening to 2 kPa [297]. Despite the differences between these studies, it emerges that the duration of mechanical memory scales with the duration of the priming period and the stiffness of the substrates used for both mechanical priming and the subsequent switch. Such a dosing effect has been systematically studied with MSCs primed for a few days on 10 kPa-stiff hydrogel substrates that allow softening to 2 kPa using a light reaction, while the MSCs can remain on the same surface without the need for passaging [312]. 

**Figure 4:**
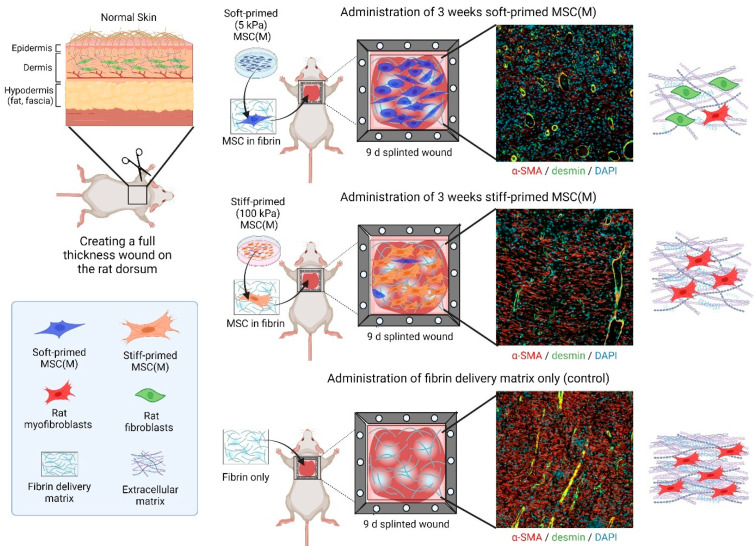
Therapeutic effects of mechanically primed MSCs on rat wound healing. Skin wound healing was the first preclinical example to show a differential effect of soft- versus stiff-primed MSCs on tissue repair after transplantation [125]. Rat bone-marrow-derived MSCs (MSC(M)), primed for 3 weeks on either soft (5 kPa) or stiff (100 kPa) silicone culture substrates, were applied in a fibrin matrix to rat skin wounds, kept open, and made hypertrophic by a plastic frame splint. Shown are immunofluorescence images of 9-day-old wound tissue cross-sections. In this experimental model, soft primed MSC(M) suppress scar features such as enhanced wound tension which is not shown in the figure but in the published work [125], myofibroblast accumulation (red only, α-SMA), high vascularization (yellow, from co-staining of vascular smooth muscle for desmin, green, and α-SMA, red), and alignment of dense collagen extracellular matrix (only shown in the schematic). All these features are enhanced after delivery of stiff-primed MSC and even further accentuated in wounds that did not receive any MSCs.

One may argue that the study of MSC mechanical priming and memory has mere academic value. However, in a clinical context, MSCs are typically expanded for 3–5 passages before enough cells are produced for a transplant, which is sufficient time to generate lasting in vitro memory. Or, in other words, build up resistance against the pro-fibrotic environment of the host wound environment. Indeed, the therapeutic potential of mechanically priming MSCs during the culture expansion phase has been shown using animal models of fibrotic healing. Rat MSC(M) directly explanted and then primed for three weeks on 5 kPa skin-soft substrates stimulated better wound healing outcomes in a rat hypertrophic model of skin wound healing compared to the delivery of 100 kPa scar-stiff-primed or culture plastic-expanded MSCs (Figure 4). Typical scar features such as poor vascularization, excessive accumulation of myofibroblasts, dense collagen, and high wound tension were all suppressed by the therapeutic soft-primed MSCs [125]. Similarly, MSCs(A) cultured on 1 kPa fat-soft substrates for 2 weeks promoted tissue regeneration after delivery into an inflammatory environment in a case of post-traumatic elbow contracture [311]. In both models, it remains to be shown whether the delivered MSCs directly contribute to the production and remodeling of wound ECM, i.e., whether their culture-acquired myofibroblast state matters. Alternatively, mechanical priming in vitro may also alter MSC secretomes and, thus, how such MSCs instruct the host inflammatory and fibroblastic cells via trophic actions in the wound bed. It also remains to be shown whether and for how long mechanical MSC memory persists after tissue delivery. 

In addition to controlling lasting fibrogenic and/or MSC immunomodulation features, in vitro mechanical priming may also be used to determine how MSCs differentiate into desired lineages upon therapeutic delivery. Repair of damaged cartilage and bone are exemplary clinical applications for MSCs, with the aim of repairing comparably stiff tissues with low regenerative capacity. For instance, MSC can be primed for osteogenesis by physiologically relevant mechanical stimuli in vitro to support bone tissue regeneration in vivo [254,313,314,315,316,317]. Thus, MSC mechanical memory can potentially be harnessed in at least two ways: first, by rendering MSCs less sensitive to stiff environments to preserve their healing potential (soft-priming) and second, by enhancing their physical response to stiff environments to guide them toward becoming osteoblasts (stiff-priming). Given that mechanical memory has only been discovered recently, pre-clinical evidence for these strategies is still scarce but is beginning to be produced.

## 5. Conclusions

The therapeutic value of donor-derived human MSCs to treat human disease conditions has been proven in numerous clinical studies. But like everything in life, (MSC) quality matters. In our review, we focused on myofibroblast activation as one specific fate that can reduce MSC quality (i.e., regenerative potential) and potentially jeopardize therapeutic success by driving fibrosis in the recipient tissue. We developed how mechanical factors—in particular the conventionally stiff culture surfaces used to expand therapeutic MSC populations—will drive MSC-to-myofibroblast activation. Chemically interfering with the discussed acute MSC mechanosensing and transduction mechanisms is one possibility to keep MSCs non-fibrogenic before transplantation. However, such treatments will not protect MSCs from myofibroblast activation in the recipient tissue, which is frequently characterized by a wound and/or fibrotic environment. We propose that persistent suppression of myofibroblast features, at least for a few days of MSC grafting, can be achieved by preconditioning (priming) MSCs on soft culture surfaces, which imprints lasting mechanical memory. Conversely, stiff mechanical priming is a possible strategy to maintain in vitro-induced osteogenic features for cartilage and bone repair applications even after delivery in conditions where the formation of these connective tissues is impaired. The advantages of tuning the mechanical cell culture environment are the simplicity of the approach and the lack of chemical interference. Both advantages are also important regulatory considerations to produce MSCs for clinical therapies.

## 6. Outlook and Future Perspectives

While animal models and clinical trials have demonstrated promising effects of MSC delivery to treat some disease conditions, consistency—and thus predictability—is often a challenge. Part of this variability is due to the heterogeneity of MSCs at the population level and their plasticity at the single-cell level. Another reason for variable therapeutic outcomes is that the immunomodulatory and regenerative properties of MSCs are often not sustained following transplantation, which raises safety and efficiency concerns for clinical applications [41,158,159,163]. One option to standardize the production of desired and more homogeneous therapeutic cells is deriving MSCs from induced pluripotent stem cells (iPSCs) [318,319,320,321]. However, mass production of iPSC-MSC is still in development, and the generation of donor-derived iPSCs adds an additional time constraint that many patients may not have the luxury to endure. 

Imprinting desired functional MSC features epigenetically during the pivotal cell culture expansion is another strategy that has great potential to enhance MSC performance after transplantation [85,322]. As an alternative to cell culture priming in the defined mechanical conditions discussed above, mechanically induced epigenetic memory is possibly altered or erased by targeting the molecular mechanisms of memory formation. Above, we have already discussed acute mechanosensitive transcription factors and epigenetic modifiers that are involved in the acquisition and maintenance of mechanically induced MSC memory, including MRTF-A/MLK-1 [125], YAP/TAZ [125,301,311,312,323], miR-21 [125], HATs, and HDACs [301] (Figure 3). While not all studies establish a direct connection between the mechanical environment of cultured plastic dishes and epigenetic memory, manipulating epigenetic modifications of conventionally cultured MSCs, such as DNA methylation, was shown to enhance their immunomodulatory and regenerative capabilities [324,325]. In our own studies, knocking down the myofibroblast memory keeper miR-21 restored the ability of 3-week stiff-primed MSCs to lose myofibroblast features and regain regenerative capabilities after a subsequent switch to soft substrates. Such stiff memory-erased MSCs improved the healing of hypertrophic rat wounds, such as 3-week soft-primed MSCs [125]. 

Inversely, experimentally increasing the global levels of histone acetylation in soft-primed MSCs created phenotypic and functional features characteristic of stiff-primed MSCs [254], including reduced multi-lineage differentiation capacity [326], apoptosis, and senescence [327]. It is tempting to try experimentally decreasing histone acetylation as another promising avenue to maintain MSCs with ‘soft skills’ even in stiff conventional culture. In addition to manipulating mechanically induced epigenetic changes at the level of DNA modifications, changes in epitranscriptomics; for instance, RNA methylation, presents an exciting new field to guide persistent MSC behavior. For instance, it has been shown that cytoskeletal changes in response to physical cues play a role in regulating the movement and localization of different RNA species and even that of ribosomes [328]. It will be worthwhile to investigate in the future how physical signals can influence MSC phenotypes through two vital aspects of protein regulation: translation and chemical modification of RNA. 

## Figures and Tables

**Table 1:** MSC Markers. Summarized are the different markers used to discriminate MSCs from other cell populations and to identify different MSC sub-populations. For markers that are commonly used in flow cytometry, we provide the cluster of differentiation (CD) nomenclature in the column ‘Marker’. For markers that are predominantly used in gene sequencing studies, we provide the respective gene symbol. References to the studies using these markers are given in the text.

	MSC Marker	Protein Name, *Gene Symbol*
*MSC Markers and Exclusion Criteria*		
MSC-‘specific’ Markers (Flow Cytometry)	CD29	β1 integrin, *ITGB1*
	CD44	Hyaluronan Receptor, *CD44*
	CD90	Thy-1, *THY1*
	CD73	Ecto-5′-nucleotidase, *NT5E*
	CD105	Endoglin-1, *ENG*
	CD36	A Receptor for Thrombospondin-1, *CD36*
	CD271	Nerve Growth Factor Receptor, *NGFR*
	CD200	CD200, *CD200*
	CD273	Programmed Cell Death 1 Ligand 2, *PDCD1LG2*
	CD274	Programmed death-ligand 1, *PDL1*
	CD146	Melanoma Cell Adhesion Molecule, *MCAM*
	CD248	Endosialin, *CD248*
	CD140B	Platelet Derived Growth Factor Receptor Beta, *PDGFRB*
MSC Exclusion Markers (Flow Cytometry)	CD31	Platelet endothelial cell adhesion molecule-1, *PECAM-1*
	CD11b	Integrin α-M, *ITGAM*
	CD14	CD14 Antigen, *CD14*
	CD19	CD19 Antigen, *CD19*
	CD34	CD34 Antigen, *CD34*
	CD45	Protein Tyrosine Phosphatase Receptor Type C, *PTPRC*
	CD79α	Immunoglobulin (Ig)α, *CD79A*
	CD117	Tyrosine-protein kinase KIT, *KIT*
	CD80	T-Lymphocyte Activation Antigen CD80, *CD80*
	CD86	T-Lymphocyte Activation Antigen CD86, *CD86*
*MSC Subpopulation-Specific Markers*		
Genes used to cluster MSCs in mouse scRNA sequencing studies	*Cd24a*	CD24a Antigen (Small Cell Lung Carcinoma Cluster 4 Antigen)
	*Entpd1*	Ectonucleoside Triphosphate Diphosphohydrolase 1, CD39
	*Icam1*	Intercellular Cell Adhesion Molecule 1, CD54
	*Il1r2*	Interleukin 1 Receptor Type 2, CD121b
	*Ly6a*	Stem Cell Antigen-1 (Sca-1)/Lymphocyte Activation Protein-6a
	*Ly6c1*	Lymphocyte Antigen 6 Family Member C1
Adipogenic MSCs	*ADIPOQ*	Adiponectin, C1Q And Collagen Domain Containing
	*MGP*	Matrix Gla protein
	*MAFF*	MAF BZIP Transcription Factor F
	*PPARG*	Peroxisome Proliferator Activated Receptor Gamma
	*CEBPB*	CCAAT Enhancer Binding Protein Beta
	*EBF2*	EBF Transcription Factor 2
	*HMGA2*	High Mobility Group AT-Hook 2
Chondrogenic MSCs	*APOD*	apolipoprotein D
	*TRPS1*	Transcriptional Repressor GATA Binding 1
	*SCX*	Scleraxis BHLH Transcription Facto
	*COL11A1*	Collagen Type XI Alpha 1 Chain
Chondrogenic & Osteogenic MSCs	*ASPN*	Asporin
	*OMD*	Osteomodulin
	*GPM6B*	Glycoprotein M6B
	*IFITM1*	Interferon Induced Transmembrane Protein 1
	*GPNMB*	Glycoprotein Nonmetastatic Melanoma Protein B
Osteogenic MSCs	*ALPL*	Alkaline Phosphatase
	*COL1a1*	Collagen Type 1
	*MCAM*	Melanoma Cell Adhesion Molecule, CD146
	*SP7*	Sp7 Transcription Factor
	*Creb3l3*	CAMP Responsive Element Binding Protein 3 Like 3
	*MEF2c*	Myocyte Enhancer Factor 2C
	*RUNX2*	RUNX Family Transcription Factor 2
	*JUN*	AP-1 Transcription Factor Subunit
	*ATF4*	Activating Transcription Factor 4
	*ID4*	Inhibitor Of DNA Binding 4
Osteogenic & Immunomodulatory MSCs	*CMKLR1*	Chemokine-Like Receptor 1
Immunomodulatory MSCs	*CD106*	Vascular Cell Adhesion Molecule 1, CD106
	*CD47*	Leukocyte Surface Antigen CD47
	*CD248*	Endosialin, CD248
	*PLAUR*	Plasminogen Activator, Urokinase Receptor, CD87
MSCs with Stemness Characteristics	*SOX4*	SRY-Box Transcription Factor 4
	*DPP4*	Dipeptidyl Peptidase 4, CD26
	*GAS1*	Growth Arrest-Specific Protein 1
	*TOP2A*	DNA Topoisomerase II Alpha
	*MKI67*	Marker Of Proliferation Ki-67
	*E2F1*	E2F Transcription Factor 1
	*E2F8*	E2F Transcription Factor 8
	*CCNA2*	Cyclin A2
	*CTCF*	CCCTC-Binding Factor
	*PBX3*	Pre-B-Cell Leukemia Transcription Factor 3
	*MYBL2*	MYB Proto-Oncogene Like 2

**Table 2:** Therapeutic MSCs in clinical trials for burn wound applications.

NCT Number	Study Title	Disease Condition	Study Aims	Phases	Sex-Age
NCT06122532	Umbilical Cord MSCs for the Repair of Large Area Burn Wounds	Large Area Burns	This study aims to utilize a prospective, open, and randomized controlled research design to investigate the efficacy and safety of employing human umbilical cord MSCs for the treatment of extensive burn injuries. Its objective is to overcome existing treatment constraints, investigate innovative clinical interventions, facilitate skin lesion repair, and enhance patient outcomes in terms of cure rates and quality of life.	not available	ALL-ADULT, OLDER_ADULT
NCT05078385	Safety of Extracellular Vesicles for Burn Wounds	Burns	Treatment of patients with deep second-degree burns of the skin with extracellular vesicles isolated from MSCs(M).	PHASE1	ALL-ADULT, OLDER_ADULT
NCT06103409	MSCs for the Treatment of Burn Wounds	Second- or Third-degree Burn Wounds	The goal of this study is to evaluate the capacity of allogenic MSC(M) or MSC(A) to induce wound healing in patients with burn wounds.	PHASE1PHASE2	All-CHILD, ADULT, OLDER_ADULT
NCT03686449	Autologous Keratinocyte Suspension Versus Adipose-Derived Stem Cell-Keratinocyte Suspension for Post-Burn Raw Area	Burn With Full-Thickness Skin Loss	Two study aims: Assess the efficiency of non-cultured autologous keratinocyte suspension in treating post-burn raw areas. Compare the results of keratinocyte suspension alone versus Adipose-derived MSCs-keratinocyte suspension in post-burn raw areas.	not available	ADULT, OLDER_ADULT
NCT03967275	Subconjunctival Injection of Allogeneic MSCs in Severe Ocular Chemical Burn	Ocular Chemical Burns	To showcase the reliability of producing MSC(M) for treating severe eye burns. Bone marrow samples, separate from those designated for transplantation, will be collected from willing donors. A maximum of three donors contribute to the production of allogeneic MSC(M), with the resulting suspension stored for 10 years to assess the stability of cryopreserved cells.	Preclinical	ALL, ADULT, OLDER_ADULT
NCT02325843	Treatment of Human Bone Marrow MSCs in Ocular Corneal Burn	Chemical Burns	To evaluate the safety and effectiveness of MSC therapy in treating corneal burns in humans. Ocular chemical burns contribute to vision loss in our country, with limited effective treatments available. Initial findings in rats with corneal alkali injuries indicated that MSCs expedited corneal healing and suppressed abnormal blood vessel formation.	PHASE2	ALL-ADULT, OLDER_ADULT
NCT04235296	MSC Conditioned Medium-derived Pleiotropic Factor in Treating Residual Burn Wound	Residual Burn Wounds	To evaluate the safety and effectiveness of MSC-released biological factors (conditioned medium-derived pleiotropic factor) to aid tissue repair.	PHASE1	ALL-CHILD, ADULT
NCT03237442	Umbilical Cord MSCs Injection for Ocular Corneal Burn	Ocular Corneal Burn	To evaluate the efficacy and safety of MSC(WJ) in treating corneal burns in humans. Chemical burns in the eye contribute to vision loss in China, with limited effective treatments available. Initial research in rabbits with corneal alkali injuries demonstrated that human MSC(WJ) accelerated corneal healing and inhibited abnormal blood vessel growth.	PHASE1, PHASE2	All-ADULT
NCT02104713	Stem Cell Therapy to Improve Burn Wound Healing	Skin Second Degree Burns	To assess the safety and effectiveness of allogeneic stem cell therapy from healthy donors in treating second-degree burn wounds covering less than 20% of the body. Phase 1 will determine safe dosage levels, followed by an expanded trial to evaluate efficacy.	PHASE1	All-ADULT, OLDER_ADULT
NCT02619851	A Clinical Trial to Evaluate the Safety and Efficacy of ALLO-ASC-DFU for Second Deep Degree Burn Injury Subjects	Burns	To test the efficacy and safety of ALLO-ASC-DFU and conventional therapy in deep second-degree burn wound subjects.	PHASE2	ALL-ADULT, OLDER_ADULT
NCT01443689	Allogenic Stem Cell Therapy in Patients With Acute Burn	Burns	To assess the safety and effectiveness of transplanting human MSC(WJ) and mononuclear cells (hCBMNCs) in patients with acute burns, offering potential advancements in burn treatment. Stem cell therapy, particularly involving MSC(WJ) and CBMNCs, shows promise in modulating immune responses and the enhancement of angiogenesis and promoting tissue repair.	PHASE1, PHASE2	ALL-ADULT, OLDER_ADULT
NCT03113747	Allogeneic ADSCs and Platelet-Poor Plasma Fibrin Hydrogel to Treat Patients With Burn Wounds (ADSCs-BWs)	Second- or Third-Degree Burns	To assess the safety and effectiveness of a tissue-engineered construct utilizing allogeneic cultured MSC(A) and platelet-poor plasma fibrin hydrogel for treating patients with second- and third-degree burn injuries.	PHASE1, PHASE2	ALL-ADULT, OLDER_ADULT
NCT02394873	A Study to Evaluate the Safety of ALLO-ASC-DFU in the Subjects With Deep Second-degree Burn Wound	Burns	To assess the safety of ALLO-ASC-DFU, a hydrogel sheet containing allogeneic adipose-derived MSCs, for treating deep second-degree burn wounds. These stem cells release growth factors such as VEGF and HGF, which can promote wound healing and tissue regeneration, potentially offering a novel treatment option for burns.	PHASE1	ALL-ADULT, OLDER_ADULT
NCT02672280	Safety and Exploratory Efficacy Study of Collagen Membrane With MSCs in the Treatment of Skin Defects	Wounds, Diabetic Foot Ulcers, Burns	To evaluate the safety and exploratory efficacy of the medical collagen membrane with MSC(WJ) in the treatment of patients with skin defects.	PHASE1, PHASE2	ALL-ADULT, OLDER_ADULT
NCT05984628	Umbilical Cord Stem Cells for Skin Grafts in Donor Site Wounds	Skin Wound, Hypertrophic Scars	This clinical trial aims to evaluate the safety and effectiveness of MSC(WJ) therapy in patients undergoing medium-thickness skin grafts for donor site wounds. It will investigate whether hUCMSC therapy improves healing quality and speed and reduces scar formation compared to standard treatment. Participants will have regular follow-ups to monitor wound healing and assess side effects. The effectiveness of MSC(WJ) therapy will be compared between the treatment group and a control group receiving standard treatment.	not available	ALL-ADULT

## Glossary

***Allogeneic MSCs***: MSCs that are derived from a donor of the same species but have a different genetic makeup. ***Autologous MSCs***: MSCs that are derived from the same donor that also receives the cell transplant. ***ISCT*** (International Society for Cell & Gene Therapy): An organization that provides guidelines and standards for cell and gene therapy. ***Progenitor cell***: Multipotent cell that can differentiate into different cell types, but with fewer lineage choices and less renewal capacity than stem cells. ***Transcriptome Signature***: Distinct gene expression profiles within the complete set of RNA transcripts produced by the genome, under specific conditions or in particular cell types, which can be used to identify cellular states or responses to treatments. ***Single-cell RNA sequencing (scRNA-seq)***: A comparably recent technique allowing for the analysis of cellular heterogeneity within a complex tissue or population by sequencing the RNA transcripts of each cell separately. ***Meta-analysis***: A statistical method that combines and analyzes data from multiple independent studies on the same topic to identify overall trends, increase statistical power, and provide a more comprehensive understanding of the research question. ***Granulation tissue***: Newly formed tissue that fills a wound with extracellular matrix and cells during the healing process, where the abundance of new capillaries crates a ‘granular’ appearance in histological sections. ***Secretome***: The complete set of proteins, peptides, extracellular vesicles, and other molecules that are secreted by cells into the extracellular space to perform various roles in intercellular communication, tissue homeostasis, and physiological functions. ***MicroRNA (miRNA, miR)***: Small non-coding RNA molecules that bind to messenger RNA (mRNA) and regulate cell processes by either degrading the mRNA or inhibiting protein translation. ***Mechanotransduction***: The cellular process of converting mechanical signals such as strain, pressure, and shear forces into chemical signalling events and cell responses. ***Epigenome***: The complete set of chemical (epigenetic) modifications made on DNA and histone proteins, which regulate gene expression and other genomic functions without altering the underlying DNA sequence. ***ATAC-Sequencing***: Stands for assay for transposase-accessible chromatin with sequencing and is a technique used to study the accessibility of chromatin for regulatory proteins, such as transcription enhancers and repressors, by employing a transposase enzyme that inserts sequencing adapters into open chromatin regions.
